# Multi-factor climate change effects on insect herbivore performance

**DOI:** 10.1002/ece3.564

**Published:** 2013-04-15

**Authors:** Christoph Scherber, David J Gladbach, Karen Stevnbak, Rune Juelsborg Karsten, Inger Kappel Schmidt, Anders Michelsen, Kristian Rost Albert, Klaus Steenberg Larsen, Teis Nørgaard Mikkelsen, Claus Beier, Søren Christensen

**Affiliations:** 1 Agroecology, Department of Crop Science, Georg-August-University of GöttingenGrisebachstrasse 6, D-37077, Göttingen, Germany; 2 Terrestrial Ecology, Department of Biology, University of CopenhagenUniversitetsparken 15, 2100, København Ø, Denmark; 3 Forest and Landscape, University of CopenhagenRolighedsvej 23, 1958, Frederiksberg C, Denmark; 4 Department of Geosciences and Natural Resource Management, University of CopenhagenRolighedsvej 23, 1958, Frederiksberg, Denmark; 5 Department of Chemical and Biochemical Engineering, Technical University of DenmarkDTU, DK-2800, Kgs. Lyngby, Denmark

**Keywords:** Chrysomelidae, climaite, condensed tannins, FACE experiment, multiple climate change drivers, multitrophic interactions, plant secondary metabolites

## Abstract

The impact of climate change on herbivorous insects can have far-reaching consequences for ecosystem processes. However, experiments investigating the combined effects of multiple climate change drivers on herbivorous insects are scarce. We independently manipulated three climate change drivers (CO_2_, warming, drought) in a Danish heathland ecosystem. The experiment was established in 2005 as a full factorial split-plot with 6 blocks × 2 levels of CO_2_ × 2 levels of warming × 2 levels of drought = 48 plots. In 2008, we exposed 432 larvae (*n* = 9 per plot) of the heather beetle (*Lochmaea suturalis* Thomson), an important herbivore on heather, to ambient versus elevated drought, temperature, and CO_2_ (plus all combinations) for 5 weeks. Larval weight and survival were highest under ambient conditions and decreased significantly with the number of climate change drivers. Weight was lowest under the drought treatment, and there was a three-way interaction between time, CO_2_, and drought. Survival was lowest when drought, warming, and elevated CO_2_ were combined. Effects of climate change drivers depended on other co-acting factors and were mediated by changes in plant secondary compounds, nitrogen, and water content. Overall, drought was the most important factor for this insect herbivore. Our study shows that weight and survival of insect herbivores may decline under future climate. The complexity of insect herbivore responses increases with the number of combined climate change drivers.

## Introduction

Herbivorous insects account for about one quarter of all extant organisms (Strong et al. 1984) and are essential to ecosystem structure and functioning (Weisser and Siemann 2004). Ecosystem process rates such as herbivory may be altered significantly under climate change (Cornelissen 2011). The global mean surface air temperature is expected to increase by 1.8–5.8°C (2090–2099 relative to 1980–1999), with additional changes in other climate change drivers such as increasing CO_2_ levels or extreme weather events (IPCC 2007). In recent studies, effects of global change drivers on herbivorous insects have mostly been studied in single-factor manipulative experiments rather than multi-factorially (Rustad 2008). For example, studies have shown that increases in CO_2_ (Stiling and Cornelissen 2007), air temperature (Bale et al. 2002) or altered water conditions (Jamieson et al. 2012) may affect plant-insect herbivore interactions. However, important interactive effects may be missed when global change drivers are applied individually. Consequently, an increasing number of studies now manipulate multiple climate change drivers at once (e.g., Shaw et al. 2002; Pritchard et al. 2007; Robinson et al. 2012; Stevnbak et al. 2012). This “next generation” of global change experiments will allow to test whether the effects of climate change drivers add up or cancel out in combination (Larsen et al. 2011; Leuzinger et al. 2011).

In this study, we independently manipulated atmospheric CO_2_ concentration, near-surface air temperature and summer drought in a replicated field experiment (Mikkelsen et al. 2008). The experiments were conducted in nutrient-poor heather vegetation dominated by *Calluna vulgaris* (L.) and *Deschampsia flexuosa* (L.) Trin. We recorded weight and survival of larvae of an important specialist herbivore, the heather beetle *Lochmaea suturalis* Thomson (Chrysomelidae). The heather beetle is a major threat to heathlands worldwide, because its population sizes periodically reach outbreak densities, causing severe and large-scale defoliations to heather. Experimental studies have suggested that warming may stimulate defoliation by heather beetles (Peñuelas et al. 2004).

Here, we measured the response of insect individuals on heather to multiple climate change effects under field conditions. Responses to climate change may be direct or plant-mediated (Cornelissen 2011; De Lucia et al. 2012). In particular, elevated CO_2_ may alter the chemical composition of plants (Peñuelas and Estiarte 1998; Awmack and Leather 2002) and thus reduce the nutritive value for herbivores (Cornelissen 2011). We therefore also assessed climate change effects on plant chemistry, in addition to our insect herbivore measurements. We tested the following hypotheses:

1. Elevated atmospheric CO_2_-concentrations negatively affect growth and survival of *L. suturalis* larvae, because plants contain more Carbon-based secondary metabolites (De Lucia et al. 2012) and less nitrogen (Stiling and Cornelissen 2007; Cornelissen 2011).
2. Prolonged drought negatively affects plant quality and hence reduces larval growth and survival (Huberty and Denno 2004).
3. Warming positively affects larval growth and survival due to higher metabolic rates (Bale et al. 2002; Jamieson et al. 2012).
4. Climate change affects herbivores both directly via physiological responses and indirectly via changes in nitrogen content and plant secondary compounds (De Lucia et al. 2012).
5. With increasing number of climate change drivers, the magnitudes of responses will decline (Leuzinger et al. 2011), that is, growth and survival will be most severely affected by single climate change drivers.

## Materials and Methods

### Site description

The experiment was conducted at the Climaite research site at Brandbjerg (55°53′N, 11°58′E), Denmark (Mikkelsen et al. 2008). The site is located on nutrient-poor sandy soils with an unmanaged dry heath/grassland mosaic consisting of heather shrubs (*C. vulgaris*, 30% cover) and grasses (*D. flexuosa*, 70% cover). The long-term annual mean air temperature was 7.7°C and the precipitation averaged 712 mm (Danish Meteorological Institute, 2013).

### Experimental design and treatments

**Figure 1:**
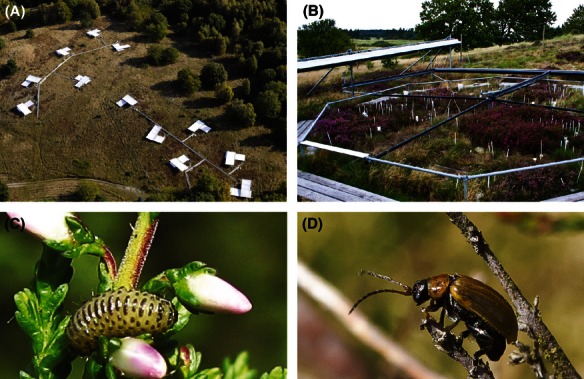
Study site and study organism. (A) Aerial view of the Climaite experiment, showing the 12 octagons with drought and warming curtains in action. Curtains were drawn over plots for illustrative purposes only. (B) A single octagon, surrounded by CO_2_ tubes, split into four subsectors where factorial combinations of drought and warming were applied. (C) Larva and (D) imago of the heather beetle, *Lochmaea suturalis* (Thomson, 1866) feeding on *Calluna vulgaris* L. (Ericaceae). Image credits: (A) T. N. Mikkelsen; (B) D. Gladbach; (C) and (D) C. Scherber.

The study was laid out as a full factorial experiment combining the effects warming (T), drought (D) and elevated CO_2_ (CO_2_) to mimic possible future climate scenarios in Denmark. Climate manipulations (T, D, CO_2_), an ambient control (A) and all combinations of them (TD, TCO2, DCO2, and TDCO2) were established in October 2005. The study site was divided into six blocks to account for potential abiotic differences. Each block consisted of two octagons (6.8 m diameter) where atmospheric CO_2_ concentration was enhanced in one of them. Each octagon was further divided into four plots (split-plot design), yielding a total of 48 plots (Fig. 1). CO_2_ was manipulated at the octagon level, while drought, elevated temperature, a combination of both treatments and a plot without drought or warming were applied within octagons (Fig. 1A, B).

Daytime air concentration of CO_2_ was elevated by ∼130 ppm using a free air carbon dioxide enrichment (FACE) system (Miglietta et al. 2001). Note that CO_2_ fumigation took place only during the daytime when plants had active uptake. Passive night time warming (Beier et al. 2004) with curtains (height 50 cm) covering the vegetation from sunset to sunrise increased the average nighttime temperature (at 20 cm above ground surface) by 1.0°C in the period from 2006 to 2008, ranging from a mean increase of 0.47°C during winter (December–February) to 1.45°C during spring/early summer (April–June). Warming curtains were withdrawn under rainfall. The drought treatment was implemented using curtains (controlled by rain sensors) that were activated once or twice a year for a period of ∼1 month. In this way, 35–76 mm of precipitation were excluded in drought plots, corresponding to 5–11% of annual precipitation (2006–2008). In the study year (2008), the drought curtains were activated from 5 to 26 May and from 16 September to 1 October. Because soils were already relatively dry when the drought was initiated in May, the treatment was stopped in late May due to very low levels of soil water content in the drought plots. Still, because only 13 mm of precipitation had been excluded, an additional drought campaign was conducted in September removing another 22 mm of precipitation this year. For further detailed information on the facility, treatments and the experimental design, see Mikkelsen et al. 2008.

### Study organism and measurements

The heather beetle (Fig. 1C, D) is a strictly monophagous insect herbivore whose larvae and adults feed on *C. vulgaris* (Mohr 1966). Outbreaks have been reported from northern Europe (Gimingham 1972), where larvae of *L. suturalis* can reach densities of up to 2000 individuals/m^2^ and cause complete defoliation and death of heather (Brunsting 1982).

On 4th May 2008, 300 adults of *L. suturalis* were caught shortly after mating in a *Calluna* heathland near Großalmerode (Germany, 55°15′N, 9°47′E). All beetles were transferred to 6-L plastic boxes (“Faunabox”, 27 × 18 × 18 cm, Savic, Belgium), where females were allowed to oviposit on moist filter paper according to standard protocols (Melber 1989). Egg batches were transferred to petri dishes (10 cm diameter), where the larvae hatched after 6–11 days and were fed on small pieces of fresh *Calluna* branches. On 30th May 2008, we installed gauze mesh bags (length 30 cm, diameter 13 cm) around heather twigs at the field site in Denmark (Fig. 1). Each plot (*N* = 48) received two mesh bags that were installed block-wise. Subplot areas receiving bags were selected at random, and heather plants within each area were selected to be of similar size and at a minimum distance of 30 cm to the plot edge. The size of each heather twig was measured using a graph paper, and every twig was digitally photographed for documentation. Subsequently, a total of 900 beetle larvae were individually weighed and grouped into three size classes. On 1st June 2008, each of the 48 “herbivory” mesh bags received the same amount of larvae from each weight class (9 individuals per bag), and initial weight of all individuals was noted. The other 48 mesh bags served as an empty control. To monitor survival and weight, larvae were recollected on days 3, 7, 16, 21, and 39 after 1st June from all four mesh bags within each octagon, counted, weighed, and returned to the plants before proceeding to the next octagon. This practice minimized the time during which the larvae were separated from the plants. Survivorship for each individual and observation day was coded as 1 (dead) or 0 (alive). Individuals lost received a censoring indicator of 0 for later survival analysis (right-censoring; Crawley 2007). Larvae were weighed used a Mettler AJ100 fine scale (Mettler-Toledo International Inc., Greifensee, Switzerland) accurate to 0.1 mg, placed on a granite block inside the local field station. Measures of fresh weight and survival were recorded weekly during larval development. The experiment was terminated when larvae were close to pupation in the litter layer (10th July 2008, i.e., after 5 weeks). All larvae alive by the end of the experiment were recollected using a pooter and kept in a freezer for further analyses.

To study plant-mediated effects, we additionally measured plant chemistry and leaf water content. In June 2007, we measured carbon (C) and nitrogen (N) content in leaves of *C. vulgaris* individuals selected at random in all 48 plots using a CN analyzer (Eurovector, Milan, Italy) coupled to an isotope ratio mass spectrometer (Isoprime, Cheadle Hulme, U.K.; for details, see Albert et al. 2011). For analysis of condensed tannins, each 200 mg of ground sample material was extracted with 10 mL absolute methanol for 20 min and centrifuged for 10 min at 3000 rpm; the supernatant was used for further analyses. Analyses were done using a vanillin bioassay (Price et al. 1978) with Catechin as a standard. Leaf water content was measured as gravimetric water content after oven-drying at 80°C.

The efficiency of climate change manipulations was assessed using (i) CO_2_ sensors, (ii) time domain reflectometry (TDR) probes for soil water content, and (iii) temperature sensors, as described in Mikkelsen et al. 2008.

### Statistical analyses

All data were analyzed using linear mixed-effects models (nlme package, version 3.1-105, date: 24 September 2012; Pinheiro et al. 2012) in R 2.15.2 (R Core Team 2012). Models contained fixed effects for warming, drought, and CO_2_ treatment (up to three-way interaction) and random intercepts for blocks (1–6), CO_2_ (0 or 1) within block and subplots (1–4) within CO_2_. For repeated-measures data, we included correlation structures (see below). Heteroscedasticity was accounted for by employing variance functions (Pinheiro and Bates 2000; Zuur et al. 2009). Initial models were fit using restricted maximum likelihood (REML) until Akaike's Information Criterion, corrected for small sample sizes (AICc) values (Burnham and Anderson 2004) indicated optimal variance, correlation, and random effects structures. We then re-fitted each model using maximum likelihood for further model simplification, employing a modified version of the stepAIC function (Venables and Ripley 2002), corrected for small sample sizes (Burnham and Anderson 2004). Final models were fitted using REML.

Effects of CO_2_ treatment on CO_2_ concentrations (1st June–10th July; 40 days, *N* = 360) were analyzed using mixed models with random slopes for date, random intercepts for blocks, an autoregressive correlation structure of order 1, and different standard deviations per octagon, with date and CO_2_ treatment as fixed effects. Only 9 octagons were used for these analyses, because control sensors in blocks 1, 3, and 6 were dysfunctional.

Mixed models on night-time temperatures at 20-cm height (1st June–10th July; 40 days, 48 plots, *N* = 1920) included polynomial random slopes (order 3) for date and an autoregressive correlation structure of order 1; the fixed effects terms were date (polynomial of order 3) in interaction with warming treatment. Soil water content at 20-cm depth (1st April–10th July; 101 days, *N* = 4848) was logit-transformed and analyzed using the same model as used for the temperature data.

The weights of individual beetle larvae were averaged for each plot and week (including the initial weights at week 0). The 5th week was excluded from weight analyses because of high larval mortality to keep the design balanced. This resulted in *N* = 48 × 5 = 240 data points (weeks 0–4) containing 49 missing values for weight. The order of fixed effect terms in maximal models was time (in weeks; polynomial of order 2), CO_2_, drought, temperature, plus two- and three-way interactions between all terms. The corresponding denominator degrees of freedom for the fixed effects in maximal statistical models were 6-1 = 5 (CO_2_), 48-6-6-5-1 = 30 (Drought × Warming, and interactions with CO_2_) and 129 (within-groups). Because plots were visited repeatedly over time, we included random slopes for weeks. Temporal autocorrelation was modeled using a first-order autoregressive correlation structure. Because the variance increased with time, time was included as a variance covariate.

The dataset for beetle survivorship contained 240 data points (weeks 1–5) with four missing values. Survivorship status (0 or 1) and observation days were used to calculate Kaplan–Meier survivorship (*p*) for each plot (plotID) using the survfit function from the survival package (version 2.36-12, 2 March 2012) using the R code survfit(Surv(days, status)∼plotID).

For further analysis, survivorship *p* for each plotID was transformed using the empirical logit


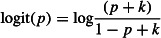
(1)

where *k* = 0.111 (the lowest observed non-zero survivorship). Predicted values were back-transformed from each fitted value 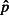 using the formula


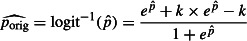
(2)

The logits of *p* were then entered as a response variable in mixed models with the fixed and random effects as described for the weight data, but with 174 within-groups degrees of freedom because all sampling weeks were included. In addition, we compared our results with mixed effects Cox proportional hazards models (coxme package, Therneau 2012).

Treatment effects on plant chemistry and leaf water content (averaged for each plot; total *N* = 48) were analyzed using mixed models with random effects for block and CO_2_ as described above.

Because larval mortality may have been size-dependent, and weight may partly have been indirectly influenced by survival, we additionally included (i) weight as a covariate in survivorship models, and (ii) survival as a covariate in weight models. These analyses showed that there were no significant inter-dependencies between weight and survival, and covariates were not retained in minimal adequate models.

In addition to the design-based models described above, we calculated a new explanatory variable, the number of climate change drivers, taking values for 0, 1, 2, or 3 climate change drivers applied. We then tested whether there was an overall effect of the number of climate change drivers on weight and survival using generalized least squares (GLS) models. GLS models showed better residual patterns and had lower AICc values than mixed models with full random effects structure. For survival data, the GLS models included a varIdent variance function (Pinheiro and Bates 2000), allowing for different variances for each number of climate change drivers.

Finally, to assess indirect effects of plant chemistry on herbivore performance, we fitted structural equation models using IBM SPSS AMOS 20.0 (IBM Corporation Software Group, Somers, NY). Initial models contained all three climate change treatments, median Kaplan–Meier survivorship *p*, and mean beetle weight. All data were scaled to [0; 1] before analysis to improve convergence of models. Two hypotheses were tested: (i) treatment effects are direct or (ii) leaf chemistry (tannin content, CN ratio) or water content (leaves, soil) indirectly mediate climate change effects. Hypothesis (ii) was tested by constructing latent variables for leaf chemistry and water content. We then used specification search and employed AIC and BIC (Bayesian Information Criterion) to arrive at the most parsimonious model.

## Results

### Efficiency of climate change treatments

**Figure 2:**
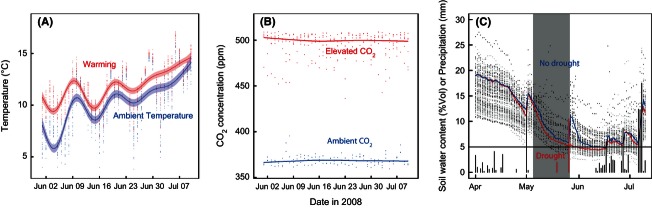
Temporal dynamics of climate change treatments applied in this experiment. (A) Nighttime air temperature at a height of 20 cm above ground in warmed (red) and unwarmed plots (blue); lines show a non-parametric smoothing spline ± 1 SE (B) Daytime air CO_2_ concentration (ppm) in elevated CO_2_ octagons (red) and ambient CO_2_ octagons (blue); lines are from a locally weighted polynomial regression smoother; (C) Soil water content (percent) from TDR probes at 0–20 cm depth in prolonged drought plots (grey) and non-drought plots (black); lines are exact mixed-effects model predictions for each day; the grey shaded area in (C) shows the period in which the drought treatment was applied. Bars in (C) show precipitation events, red bars indicate the precipitation events that were excluded in the drought treatment.

All three climate change treatments had remarkably strong and consistent effects (Fig. 2).

In the study period (1st June until 10th July 2008), CO_2_ was elevated from 360 ppm to 500 ppm on average (40 days, *N* = 360, *F*_1,352_ = 17222, *P* < 0.001).

Night temperatures were elevated by around 3.5°C (from 6.2°C to 9.7°C) at the beginning of the study period. At the end of the study period, the temperature difference was still 1.2°C (all days: *N* = 1920; Date (3rd order polynomial): *F*_3,1901_ = 247.7, *P* < 0.0001; Warming: *F*_1,1901_ = 143.74, *P* < 0.0001; Date:Warming *F*_3,1901_ = 9.16, *P* < 0.0001).

The drought treatment was applied between 5th and 26th May, which highly significantly decreased soil water content by up to 51 percent (27th May; Overall model: *N* = 101 days × 48 plots = 4848; *F*_1,4635_ = 165, *P* <0.0001): Soil water content decreased from 14.2% (4th May) to 5.3% (27th May) in drought plots, while the soil remained significantly wetter in control plots (10.3%; individual *t*-test for drought on 27th May: *t* = −8.3, df = 4635, *P* < 0.001). The drought effect remained highly significant until 5th June, that is, the natural drought was extended for a period of about 1 week; for detailed dynamics, see Figure 2. Overall, our climate manipulations consistently affected key abiotic properties (CO_2_ concentration, air temperature, and soil moisture).

### Herbivore weight

**Figure 3:**
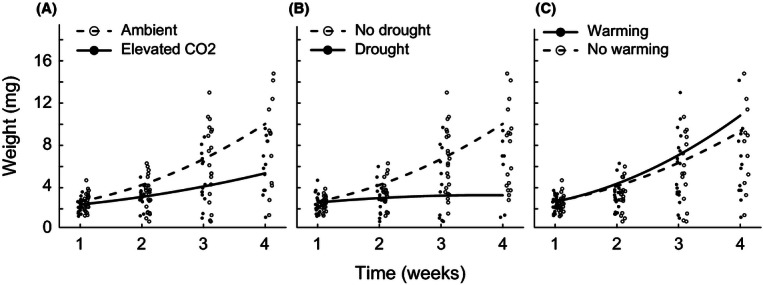
Weight (mg) of *Lochmaea suturalis* larvae over time for plots with elevated treatments (*N* = 24 per time point; filled circles, solid lines) and controls (*N* = 24 per time point; open circles, dashed lines). (A) Ambient versus Elevated CO_2_; (B) No Drought versus Drought; (C) Warming versus No Warming. Lines in (A) and (B) show model predictions from minimal adequate mixed-effects models; lines in (C) derived from a mixed model with all fixed effects terms included. The time:CO_2_ effect in (A) was significant in interaction with drought (*P* = 0.001; see Table 1); the drought:time effect in (B) had *P* = 0.003; warming (C) had no significant effect on weight.

The heather beetle larvae weighed 1.97 ± 0.03 mg (*N* = 48) at the beginning of the experiment (1st June). After 5 weeks, they had reached a final weight of 7.16 ± 0.77 mg (*N* = 24). Weight increase over time was particularly strong under ambient CO_2_ (Fig. 3A). There was a significant three-way interaction effect of time, drought, and CO_2_ on larval weight (Tables 1, S1; Fig. 5A): The CO_2_ effect increased over time, when no drought was applied; the same was true for drought: The drought effect increased over time, but only under ambient CO_2_ concentration. Hence, combinations of CO_2_ and drought changed temporal system dynamics and lead to outcomes that were more difficult to interpret. In addition to these interactive effects, larvae exposed to drought gained weight much more slowly than larvae not exposed to drought (polynomial time:drought interaction, Tables 1, S1 and Fig. 3B). While warming did not have a significant main effect on larval weight (Fig. 3C), it had a negative effect on the latent variable “water content” (Fig. 6; structural equation model, Table S2, partial slope: −0.367 ± 0.195, *P* = 0.059). Water content was also significantly reduced under drought (Table S2, partial slope: −0.830 ± 0.239, *P* < 0.001). Overall, the latent variable “water content” highly significantly negatively affected the weight of the larvae (Table S2, partial slope: 1.050 ± 0.384, *P* < 0.001). Thus, warming and drought interactively affected weight by reducing (soil) water content. Note that leaf water content appeared higher under drought/warming, because of a transient physiological response (see discussion).

**Table 1:** ANOVA tables of the minimal adequate models for weight and survival. poly(time, 2) is an orthogonal polynomial of order 2

	numDF	denDF	*F*-value	*P*-value
Response: weight (mg)				
(Intercept)	1	135	479.09	<0.0001
poly(time, 2)	2	135	31.29	<0.0001
CO_2_	1	5	3.43	0.123
DROUGHT	1	34	0.27	0.604
poly(time, 2): CO_2_	2	135	1.37	0.257
poly(time, 2):DROUGHT	2	135	5.95	0.003
CO_2_:DROUGHT	1	34	0.01	0.919
poly(time, 2): CO_2_:DROUGHT	2	135	8.08	0.001
Response: logit(survival)				
(Intercept)	1	182	7.98	0.005
poly(time, 2)	2	182	80.70	<0.0001
CO_2_	1	5	5.12	0.073
DROUGHT	1	30	12.11	0.002
TEMP	1	30	0.53	0.474
poly(time, 2): CO_2_	2	182	2.91	0.057
poly(time, 2):DROUGHT	2	182	10.56	<0.0001
CO_2_:DROUGHT	1	30	0.07	0.789
CO_2_:TEMP	1	30	3.20	0.084
DROUGHT:TEMP	1	30	0.03	0.872
CO_2_:DROUGHT:TEMP	1	30	6.19	0.019

Terms are tested in the order in which they appear in the table (principle of marginality).

Notably, combinations of all three climate change drivers decreased larval weight particularly strongly (Fig. 5A), indicating that combined drivers had stronger effects than drivers alone (for details see section on combined drivers).

### Herbivore survival

**Figure 4:**
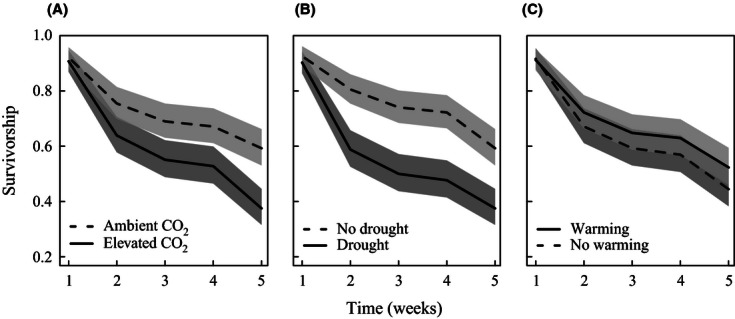
Kaplan–Meier survivorship of *Lochmaea suturalis* larvae over time for plots with elevated treatments (*N* = 24 per time point, solid lines) and ambient plots (*N* = 24 per time point, broken lines). Grey shaded areas show 95% confidence intervals of the Kaplan–Meier estimator. Significance of interactions with time: (A) *P* = 0.057; (B) *P* < 0.0001. (C) Warming was only significant in a three-way interaction with CO_2_ and drought (*P* = 0.019).

**Figure 5:**
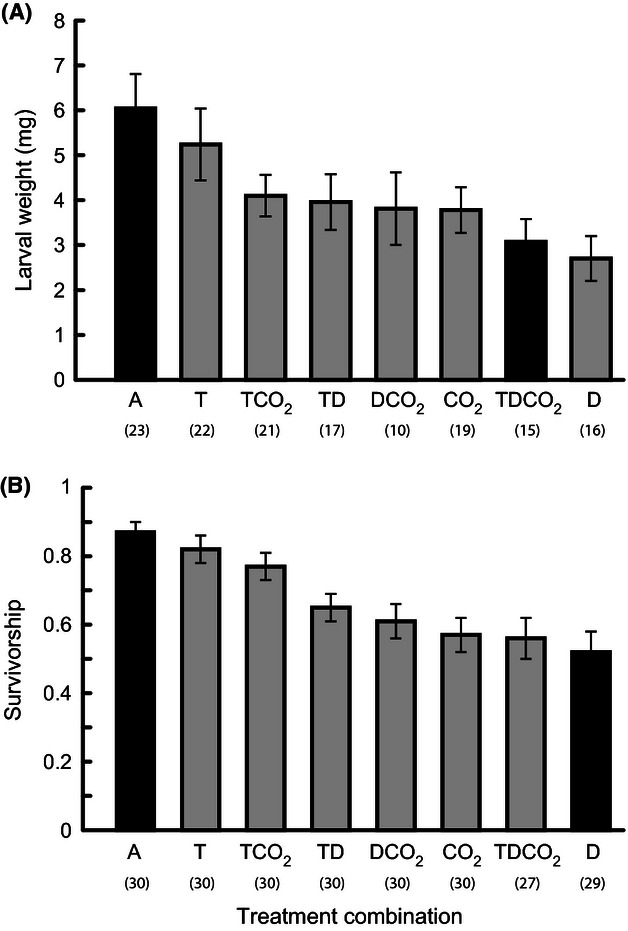
Effects of combinations of climate change treatments on (A) mean larval weight and (B) mean larval survival. Black bars indicate the two most extreme conditions applied in this experiment – either “fully ambient” or “full climate change”, that is, a combination of all three climate change treatments. Sample sizes are given in brackets.

**Figure 6:**
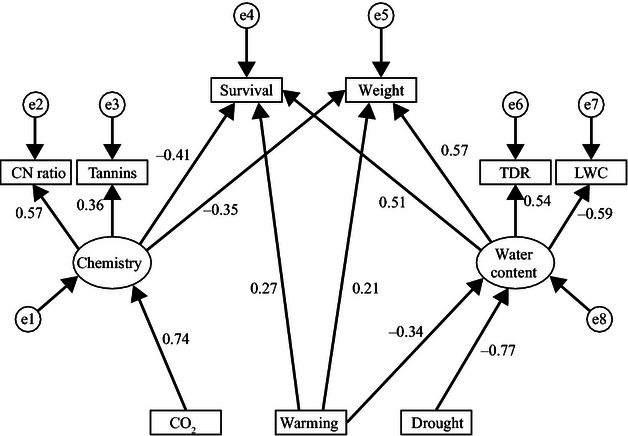
Structural equation model showing how treatment effects of CO_2_, warming and drought (bottom) affect survival and weight (top), mediated via changes in plant chemistry (latent variable) and soil/plant water content (latent variable).Variables e3-e8 are error terms. For clarity, standardized path coefficients are shown. All variables with error terms were scaled before analysis. CN, carbon/nitrogen ratio; Tannins, condensed tannins; TDR, soil water content; LWC, leaf water content.

Five weeks after the start of the experiment, an average of 4.5 ± 0.36 *L. suturalis* larvae (i.e., 50%) had survived (Table 1). Overall, survival declined non-linearly with time (Tables 1, S1; Fig. 4). However, survival was strongly and mostly negatively influenced by all three climate change drivers (Fig. 5B): First, there was a significant three-way interaction between drought, warming, and CO_2_: Warming, drought, and CO_2_ had generally negative effects on survival, but elevated CO_2_ had a positive effect in combination with drought and no warming (see Fig. 5B for more details about effects of all combinations). In addition, drought had a strongly nonlinear effect on survival over time (Table 1, Fig. 4). In structural equation models (Fig. 6), warming had a slightly positive direct effect on survival (partial slope: 0.525 ± 0.3, *P* = 0.081). The combination of all three climate change drivers decreased survival most strongly (Table S2), again indicating that the number of climate change drivers may be of importance in itself. Additional Cox mixed models had identical direction and very similar magnitudes of effects. Briefly, elevated CO_2_ increased the risk of death 3.5-fold (*P* < 0.001), drought increased the risk of death 2.6-fold (*P* < 0.01), and warming non-significantly increased the risk of death 1.2-fold (*P* = 0.6).

### Effects of plant chemistry on weight and survival

Leaf tannin content (*F*_1,5_ = 7.35, *P* = 0.042) and leaf C:N ratio (*F*_1,5_ = 13.92, *P* = 0.014) were significantly higher under elevated CO_2_ than under ambient conditions (Fig. S1). In addition, leaf water content was significantly increased under drought (*F*_1,30_ = 86.63, *P* < 0.0001) and warming (*F*_1,30_ = 15.95, *P* = 0.0004), and there was a significant interaction among CO_2_ and Drought on leaf water content (*F*_1,30_ = 5.286, *P* = 0.0286).

We tested whether these changes in plant chemistry or physiological status would also affect our invertebrate herbivore. Structural equation modeling (Fig. 6**,** Table S2) showed that the following pathways were supported: (i) indirect paths from CO_2_ to leaf chemistry to survival and weight; (ii) direct paths from elevated temperature to survival, weight, and leaf/soil water content, and (iii) an indirect path from drought via water content to weight and survival. Overall, the structural equation model had *χ*^2^ = 21, df = 23, *P* = 0.584, and the residual mean square error of approximation was within the interval [0.000; 0.108], all indicating an adequate fit between the hypothesized structural relationships and the data.

### Effects of the number of climate change drivers on weight and survival

**Figure 7:**
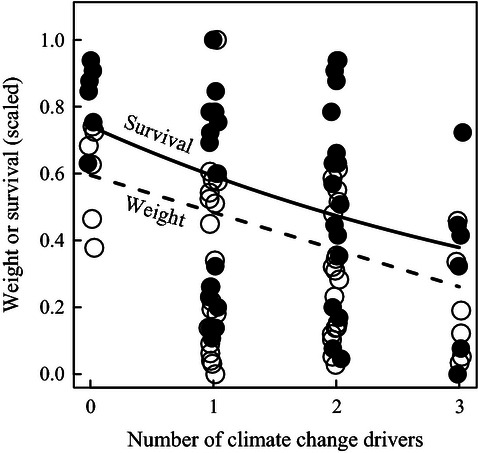
Weight (open circles) and survival (filled circles) of beetle larvae (*N* = 48) as a function of the number of climate change drivers, scaled to [0;1] using a ranging transformation. Survival (solid line) and growth (dashed line) decreased significantly (*P* < 0.05) with the number of climate change drivers. Lines show local smoothing splines (for illustrative purposes only).

Both larval weight (*F*_1,46_ = 9.12, *P* = 0.0041) and survival (*F*_1,46_ = 7.18, *P* = 0.0102) decreased significantly with the number of climate change drivers (Fig. 7). For survival, the values for the varIdent structure were 1, 2.99, 2.12, and 1.14 for 0, 1, 2, or 3 climate change drivers, indicating that variance in survivorship was maximal for 1 climate change driver and minimal for 0 or 3 climate change drivers acting simultaneously. Notably, models where the number of climate change drivers was the only explanatory variable explained the data almost as well as models containing CO_2_, Drought or their interaction (ΔAICc = 0.95 for survival; ΔAICc = 4.62 for weight).

## Discussion

We investigated main effects and interactions of the climate change drivers CO_2_, warming and drought on weight and survival of larvae of a chrysomelid beetle. As hypothesized, elevated CO_2_ (Hypothesis 1) and drought (Hypothesis 2) adversely affected weight and survival of the larvae of *L. suturalis*. Warming (Hypothesis 3) had positive or negative effects, depending on combinations with other drivers. The effects of CO_2_ and drought were clearly mediated by changes in leaf chemistry, soil, and plant water content (Hypothesis 4). Two- and three-way interactions of global change drivers in most cases amplified – not attenuated – the negative impacts of main effects on weight or survival; this finding is in contrast to other studies (e.g., Larsen et al. 2011) who found that climate change effects dampened when more factors were applied in combination. Furthermore, we have shown that the number of climate change drivers, and not just their identity, affects herbivore survival and growth (Hypothesis 5).

Of the individual drivers studied by us, drought and CO_2_ clearly had the most negative effects on herbivore performance. Our structural equation model showed that these effects were indirect and not direct. That is, our data show that CO_2_ did not kill insects directly, but acted indirectly via changes in plant chemistry. The same was true for drought: Drought (and partly also warming) acted via strong and persistent changes in soil water content and plant leaf water content. Every plot in our study had been constantly subjected to global change treatments for a period of more than 2 years; therefore, it is likely that beetle growth and survival were driven by long-term changes in plant physiological status. The adverse effects of drought on herbivore performance are perfectly in line with predictions just recently published by several authors (Cornelissen 2011; De Lucia et al. 2012).

While a reduction in soil water content from 10% to 5% may appear small, such a small difference can bring individual *Calluna* plants close to the permanent wilting point (as evident from Fig. 2C). At such low soil water content, it has been shown that stomatal conductance and photosynthesis are strongly reduced (Albert et al. 2012). Hence, the drought treatment was both statistically and biologically significant. The low soil water content in both treatment and control plots was the result of the sandy soil at the study site, in combination with a severe natural drought that occurred at the site in spring 2008 (Kongstad et al. 2012). Surprisingly, leaf water content increased under drought; this is likely a transient physiological response caused by lower stomatal conductance and higher water use efficiency (Albert et al. 2012). It is likely that the apparent anti-correlation between leaf water content and drought treatment is the result of the coupling of two non-linear processes (Sugihara et al. 2012).

While CO_2_ and drought had persistent and strong effects on herbivore performance, warming had mixed and much smaller effects. This is surprising, given our strong increase in night-time temperature of 1.2–3.5°C during our study period, from which one might have expected a 5–10% increase in mortality because of the temperature-dependence of metabolic rates (Savage et al. 2004). In our study, warming acted interactively with other drivers, indicating that our insect herbivore was more susceptible to drought and plant chemistry. This interpretation is further backed up by studies conducted by Melber (1989), showing that especially the young developmental stages of *L. suturalis* are particularly sensitive to drought, but comparatively insensitive to temperature.

The most surprising result of our study was that the number of climate change drivers in itself had a consistently negative effect on herbivore performance, independent of the identity of the climate change drivers. To our knowledge, such a result has never been reported before and is worth further investigations in other systems. In particular, previous studies have mostly reported a dampening effect of multiple climate change drivers (e.g., Leuzinger et al. 2011). Our study shows that the identity of climate change drivers matters (c.f. Fig. 5), but on top of that also the number of these drivers. An interesting analogy comes from biodiversity-ecosystem functioning research, where researchers often try to disentangle “species richness” from “species identity” effects (e.g., Crawley et al. 1999). In this study, the identity of climate change drivers (CO_2_, drought or warming) was clearly more important than the number of drivers – but it should be kept in mind that the number of drivers may act on top of individual drivers.

As every controlled experimental study, our study may be criticized for its artificiality; in particular, some of our results could have been influenced by cage effects, that is, the potentially artificial abiotic or biotic conditions inside our herbivore cages. However, the space and the amounts of heather resource contained in the 30-cm cages were beyond larval movement (few cm/day) and the material provided by far exceeded larval food consumption (Melber 1989). Gauze cages were light-transmissive, minimizing potential reductions of overall temperature by shading; treatment effects were not affected, because passive night time warming was independent of the light regime.

In addition, one may criticize our study because we only observed larvae and not the full life cycle of the heather beetle. However, insect larval stages are generally considered most sensitive to environmental changes (Zalucki et al. 2002), and this is particularly true for our study organism (Melber & Heimbach 1984; Melber 1989). Hence, if the larval stages are affected by climate change, then overall population growth may also be strongly affected, especially if climate change induces transient population dynamics (Tenhumberg 2010). Larval growth and survival can therefore be seen as indicators of potential fecundity. In the long term, combinations of climate change factors will therefore present a potent evolutionary force for insect herbivores (Coviella and Trumble 1999).

As our experimental design was essentially a crossed random effects design, critiques might argue that we should have incorporated this into our statistical analyses. We re-fitted our two main models using the lmer function in R′s lme4 library (Bates et al. 2012, version 0.999999-0) with crossed random effects for Warming and Drought nested in each CO_2_ ring. The results we obtained were essentially similar, and we thus preferred the more established “traditional” lme models, given that lme4 is still in its beta development stage. Furthermore, it was important for us to be able to account for temporal autocorrelation and variance heterogeneity, which also would not have been possible using the lmer function.

Overall, we have shown that the performance of insect herbivores may be strongly affected by drought, CO_2_, and by interactions between climate change drivers. Warming effects were generally weak. The complexity of insect responses increased with the number of combined climate change drivers. In contrast to other studies (e.g., Coley 1998; Klapweijk et al. 2010), we found no evidence for an increased insect population growth under experimental warming. Rather, our results indicate that climate change can reduce insect populations. Increasing plant C/N ratios and higher leaf tannin content may increase the duration of insect developmental stages, because nitrogen acquisition and detoxification of plant secondary compounds are more costly to herbivores. Furthermore, “extreme weather” events with prolonged drought periods may negatively affect insect herbivores, which may be aggravated by warming (Rouault et al. 2006).

Our study emphasizes that assessment and generalizations of the overall effects of future climate change based on studies of single climate change drivers should be handled with care, as the effect of one climate change driver demonstrably depends on the concert of co-acting global change drivers.

## Supporting Information

**Table S1.** Parameter estimates and standard errors from minimal adequate mixed-effects models fit by REML. The intercept shows the overall mean, all other terms are successive differences among means: Elevated-ambient CO_2_, Drought-No drought and Warming-No warming. For example, average survival was logit.e(0.71, 0.111) = 0.72.poly(time, 2)*k* is a kth-order polynomial.

**Table S2.** Partial slopes of the structural equation model presented in Figure 4.

**Figure S1.** Effects of elevated CO_2_ on leaf chemistry of *Calluna vulgaris*. Both condensed tannins (A) and C:N ratio (B) are hypothesized to influence growth and survival of insect herbivores.
