# Supplementary material for: Multi-factor climate change effects on insect herbivore performance
Source: Ecol Evol. 2013 Apr 15;3(6):1449–60. doi: 10.1002/ece3.564 (PMC3686182; doi:10.1002/ece3.564)
Supplement: Supplementary file 1 [file ece30003-1449-SD1.docx]

**Table S1** Parameter estimates and standard errors from minimal adequate mixed-effects models fit by REML. The intercept shows the overall mean, all other terms are successive differences among means: Elevated-ambient CO_2_, Drought-No drought and Warming-No warming. For example, average survival was logit.e(0.71,0.111)= 0.72. poly(time, 2)k is a k^th^-order polynomial.

| **Response: weight (mg)** | **Value** | **SE** | **DF** | **t-value** | **p-value** |
| --- | --- | --- | --- | --- | --- |
| (Intercept) | 3.36 | 0.21 | 135 | 16.01 | <0.001 |
| poly(time, 2)1 | 19.02 | 2.63 | 135 | 7.24 | <0.001 |
| poly(time, 2)2 | 4.20 | 0.95 | 135 | 4.44 | <0.001 |
| CO_2_ | -0.44 | 0.38 | 5 | -1.17 | 0.296 |
| DROUGHT | -0.83 | 0.38 | 34 | -2.17 | 0.037 |
| poly(time, 2)1: CO_2_ | -3.95 | 5.25 | 135 | -0.75 | 0.453 |
| poly(time, 2)2: CO_2_ | 0.67 | 1.89 | 135 | 0.35 | 0.725 |
| poly(time, 2)1:DROUGHT | -12.75 | 5.25 | 135 | -2.43 | 0.017 |
| poly(time, 2)2:DROUGHT | -4.42 | 1.89 | 135 | -2.34 | 0.021 |
| CO_2_:DROUGHT | 1.80 | 0.76 | 34 | 2.36 | 0.024 |
| poly(time, 2)1: CO_2_:DROUGHT | 32.51 | 10.50 | 135 | 3.10 | 0.002 |
| poly(time, 2)2: CO_2_:DROUGHT | 15.14 | 3.78 | 135 | 4.01 | <0.001 |
| **Response: logit(survival)** |  |  |  |  |  |
| (Intercept) | 0.71 | 0.18 | 182 | 4.01 | <0.001 |
| poly(time, 2)1 | -9.72 | 0.80 | 182 | -12.15 | <0.001 |
| poly(time, 2)2 | 2.17 | 0.55 | 182 | 3.98 | <0.001 |
| CO_2_ | -0.51 | 0.19 | 5 | -2.77 | 0.039 |
| DROUGHT | -0.82 | 0.19 | 30 | -4.41 | 0.000 |
| TEMP | 0.14 | 0.18 | 30 | 0.76 | 0.456 |
| poly(time, 2)1: CO_2_ | -3.50 | 1.46 | 182 | -2.40 | 0.017 |
| poly(time, 2)2: CO_2_ | 0.33 | 1.09 | 182 | 0.30 | 0.766 |
| poly(time, 2)1:DROUGHT | -3.62 | 1.46 | 182 | -2.48 | 0.014 |
| poly(time, 2)2:DROUGHT | 4.20 | 1.09 | 182 | 3.85 | <0.001 |
| CO_2_:DROUGHT | -0.10 | 0.36 | 30 | -0.27 | 0.791 |
| CO_2_:TEMP | 0.64 | 0.36 | 30 | 1.78 | 0.085 |
| DROUGHT:TEMP | -0.06 | 0.36 | 30 | -0.17 | 0.864 |
| CO_2_:DROUGHT:TEMP | -1.78 | 0.72 | 30 | -2.49 | 0.019 |

**Table S2**

Partial slopes of the structural equation model presented in Figure 4.

|  |  |  | Estimate | SE | t-value | P-value |
| --- | --- | --- | --- | --- | --- | --- |
| Chemistry | <--- | CO_2_ | 0.528 | 0.260 | 2.027 | .043 |
| Water content | <--- | Drought | -0.830 | 0.239 | -3.472 | <0.001 |
| Water content | <--- | Warming | -0.367 | 0.195 | -1.885 | .059 |
| Leaf tannin concentration | <--- | Chemistry | 1.000 |  |  |  |
| Soil water content | <--- | Water content | 1.000 |  |  |  |
| Survival | <--- | Chemistry | -1.130 | 0.667 | -1.696 | .090 |
| Weight | <--- | Water content | 1.050 | 0.384 | 2.732 | .006 |
| Weight | <--- | Chemistry | -0.977 | 0.610 | -1.602 | .109 |
| Survival | <--- | Water content | 0.929 | 0.364 | 2.554 | .011 |
| Leaf C:N ratio | <--- | Chemistry | 1.582 | 0.861 | 1.838 | .066 |
| Leaf water content | <--- | Water content | -1.085 | 0.373 | -2.907 | .004 |
| Survival | <--- | Warming | 0.525 | 0.300 | 1.747 | .081 |
| Weight | <--- | Warming | 0.423 | 0.309 | 1.370 | .171 |


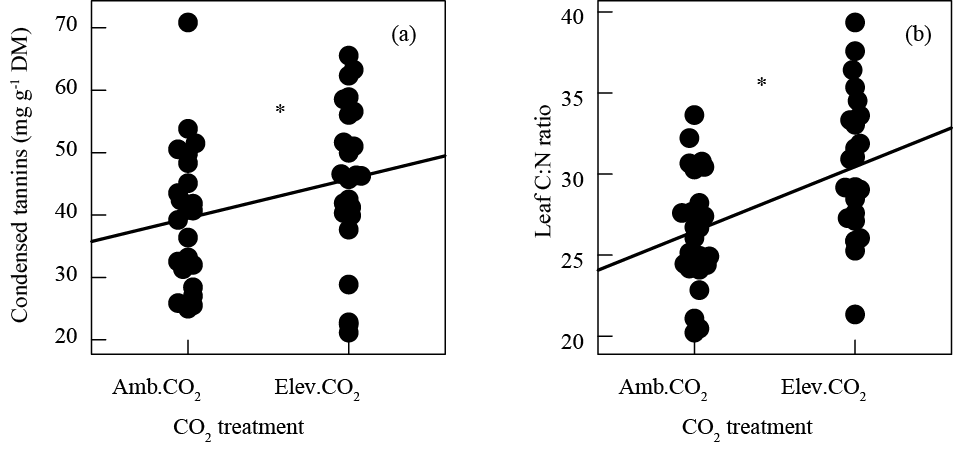


**Figure S1.** Effects of elevated CO_2_ on leaf chemistry of *Calluna vulgaris*. Both condensed tannins (a) and C:N ratio (b) are hypothesized to influence growth and survival of insect herbivores.
